# Think 500, not 50! A scalable approach to student success in STEM

**DOI:** 10.1186/s12919-017-0094-5

**Published:** 2017-12-04

**Authors:** William R. LaCourse, Kathy Lee Sutphin, Laura E. Ott, Kenneth I. Maton, Patrice McDermott, Charles Bieberich, Philip Farabaugh, Philip Rous

**Affiliations:** 1 College of Natural and Mathematical Sciences, University of Maryland, Baltimore County, Baltimore, MD 21250 USA; 2 Department of Psychology, University of Maryland, Baltimore County, Baltimore, MD 21250 USA; 3 Office of the Provost, University of Maryland, Baltimore County, Baltimore, MD 21250 USA; 4 Department of Biological Sciences, University of Maryland Baltimore County, Baltimore, MD 21250 USA

## Abstract

**Background:**

UMBC, a diverse public research university, “builds” upon its reputation in producing highly capable undergraduate scholars to create a comprehensive new model, STEM BUILD at UMBC. This program is designed to help more students develop the skills, experience and motivation to excel in science, technology, engineering, and mathematics (STEM). This article provides an in-depth description of STEM BUILD at UMBC and provides the context of this initiative within UMBC’s vision and mission.

**Key highlights:**

The STEM BUILD model targets promising STEM students who enter as freshmen or transfer students and do not qualify for significant university or other scholarship support. Of primary importance to this initiative are capacity, scalability, and institutional sustainability, as we distill the advantages and opportunities of UMBC’s successful scholars programs and expand their application to more students. The general approach is to infuse the mentoring and training process into the fabric of the undergraduate experience while fostering community, scientific identity, and resilience. At the heart of STEM BUILD at UMBC is the development of BUILD Group Research (BGR), a sequence of experiences designed to overcome the challenges that undergraduates without programmatic support often encounter (e.g., limited internship opportunities, mentorships, and research positions for which top STEM students are favored). BUILD Training Program (BTP) Trainees serve as pioneers in this initiative, which is potentially a national model for universities as they address the call to retain and graduate more students in STEM disciplines – especially those from underrepresented groups. As such, BTP is a research study using random assignment trial methodology that focuses on the scalability and eventual incorporation of successful measures into the traditional format of the academy.

**Implications:**

Critical measures to transform institutional culture include establishing an extensive STEM Living and Learning Community to increase undergraduate retention, expanding the adoption of “active learning” pedagogies to increase the efficiency of learning, and developing programs to train researchers to effectively mentor a greater portion of the student population. The overarching goal of STEM BUILD at UMBC is to retain students in STEM majors and better prepare them for post baccalaureate, graduate, or professional programs as well as careers in biomedical and behavioral research.

## Background and context

STEM Building Undergraduate Innovations in Leadership and Diversity at UMBC, or STEM BUILD at UMBC, aims to address the imperative of three contemporary reports: *Engage To Excel: Producing One Million Additional College Graduates With Degrees In Science, Technology, Engineering, and Mathematics* [1]; *Rising Above the Gathering Storm* [2]; and *Expanding Underrepresented Minority Participation* [3]. It does so by clearing the pathway for the multitude of students who enter universities and colleges with the potential to succeed, but who are not eligible for significant university or other scholarship support on the basis of their previous academic performance. Members of this highly diverse population of students (i.e., entering freshmen and transfer students) may be highly motivated to pursue STEM majors, but lack adequate support and resources to reach their goal. For example, the need to have paid employment negatively impacts students from low-income families in their ability to dedicate the time and attention needed to complete STEM degrees, particularly in the areas of biomedical or behavioral research. The 2012 report by the President’s Council of Advisors on Science and Technology (PCAST), *Engage to Excel*, identified three key reasons that students abandon STEM majors: uninspiring introductory courses, difficulties with the math required in introductory STEM courses, and an unwelcoming atmosphere from faculty in STEM courses [1].

**Fig. 1:**
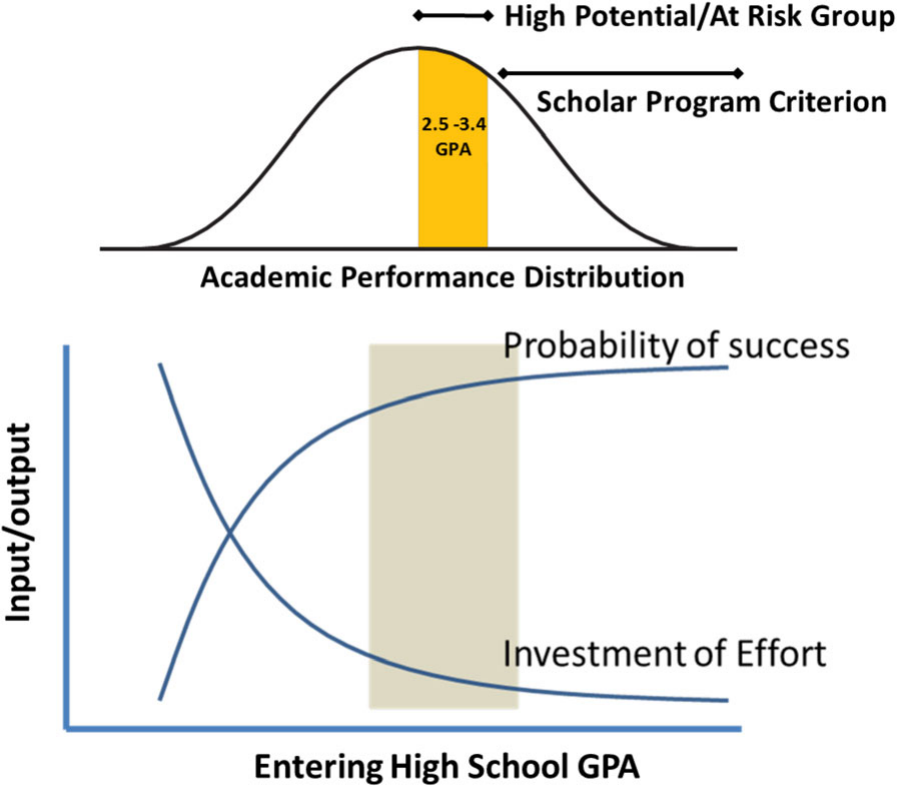
Relationship between academic performance, investment of effort by the institution, and the probability of a student’s success

STEM BUILD at UMBC is addressing these issues by establishing and studying comprehensive support strategies for a community of funded BUILD Training Program (BTP) Trainees and Affiliates. We are “building” upon UMBC’s rich experiences as a public research university in producing highly capable and competitive undergraduate scholars in a diverse setting. This initiative targets promising, entering first-time full-time (FTF) and transfer students, who are interested in pursuing STEM majors but are at risk of switching to non-STEM majors, graduating with low GPAs, or not graduating. This targeted category of *Tier 2* undergraduates who, for the purpose of this initiative, would typically be represented in the upper-middle distribution of a standard bell curve (Fig. 1). They are promising students who exceed UMBC’s admission requirements but do not qualify for significant university or other scholarship support. These Tier 2 students typically matriculate into UMBC as freshmen or transfer students with entering cumulative GPAs less than 3.4 on a maximum GPA scale of 4.0, which is the threshold for many scholars programs. UMBC’s 2003–2007 institutional cohort data (*N* = 1755) indicates that for students matriculating into targeted STEM degree programs as freshmen, 31.6% of students with final cumulative GPAs lower than 3.2 and 4.15% with cumulative GPAs greater than/equal to 3.2 did not complete a degree in 6 years. This pattern holds across all race and ethnicity categories for Tier 2 students despite their having started at UMBC with high interest in completing STEM majors. In addition, 9.85% of the students in this six-year cohort with final cumulative GPAs lower than 3.2 earned degrees in non-targeted programs in 6 years, representing an additional loss in STEM majors. The disciplines targeted in this initiative include those related to biomedical or behavioral science under the departments of biological sciences, mathematics and statistics, chemistry and biochemistry, chemical and biochemical engineering, mechanical engineering, and psychology.

### Institutional context

#### Overview of UMBC

Established in 1966, UMBC is one of 12 institutions in the University System of Maryland. At its inception, UMBC was intended to prepare students for professional schools in downtown Baltimore. UMBC’s rapid development as a public research university is reflected by its recognition and classification by the Carnegie Foundation for the Advancement of Teaching as a Research University (High Research Activity). UMBC delivers a distinctive undergraduate educational experience characterized by a strong liberal arts and sciences core, and it offers graduate programs emphasizing selected areas of science, engineering, information technology, public policy, and human services. UMBC aspires to “integrate teaching, research, and service to benefit the citizens of Maryland,” and to advance “knowledge, economic prosperity, and social justice by welcoming and inspiring inquisitive minds from all backgrounds” [4]. Students come from nearly all of the 50 states and more than 100 nations, creating a richly diverse student body and an exciting atmosphere for learning and teaching. The Fall 2016 enrollment was 13,640, which included 11,142 undergraduate and 2498 graduate students. Of the Fall 2016 undergraduates who identified a single ethnicity, 17.4% are Black, 20.9% are Asian and 6.7% are Hispanic with an overall undergraduate gender ratio of 55.4% male and 44.5% female. A significant number of our undergraduates transfer from other institutions, with about half of each year’s new students coming from community colleges and, of these, about one-third planning to pursue degrees in STEM. UMBC offers 55 majors, 35 minors and 24 certificate programs.

Inquiry is central to UMBC’s curriculum. UMBC faculty and researchers actively seek collaborative research opportunities and undergraduates are encouraged to pursue their own research questions with the support of faculty mentors. Hacker and Dreifus [5] commented on UMBC’s integration of research and instruction in their book *Higher Education:* “Of all the research universities we visited, UMBC was the place that we thought had most capably connected its research functions with its undergraduate schooling.” The University attracts high-achieving students through the nationally acclaimed Meyerhoff Scholars Program and a number of other scholars programs.

One of UMBC’s goals is to offer academically talented undergraduate students an honors university experience. We are a young university with a national footprint and reputation that receives continuous recognition for its 1) strong commitment to undergraduate teaching and innovation (U.S. News and World Report); 2) strong research, innovation, and international outlook (Times Higher Education); and 3) “Best Value” (The Princeton Review, Kiplinger’s Personal Finance, and Fiske Guide to Colleges).

### History of UMBC student research training programs

Over the past 26 years, the Meyerhoff Scholars Program has received national attention for graduating hundreds of underrepresented minority students, most of whom have gone on to pursue graduate degrees [6] and successful careers. Research shows that Meyerhoff Scholars are twice as likely to earn a STEM bachelor’s degree and more than five times more likely to enroll in and complete graduate study compared to students who were accepted but declined to participate in the Meyerhoff Scholars Program [7–10]. Further, the sense of community developed as part of the Meyerhoff Scholars Program, especially at the end of the first year, correlates to increased science identity and research self-efficacy [10]. As a result of the Meyerhoff Scholars Program and other initiatives focused on minority student achievement in STEM, UMBC has become a national leader in the number of African American bachelor’s degree recipients who go on to complete a Ph.D. in STEM fields, particularly in the life sciences [9–11]. Similarly, the Maximizing Access to Research Careers Undergraduate Student Training in Academic Research (MARC U*STAR), the Howard Hughes Medical Institute Undergraduate Scholars, and the University System of Maryland’s Louis Stokes Alliance for Minority Participation (LSAMP) Programs have increased the quantity and quality of underrepresented minority and other students receiving baccalaureate degrees in STEM fields as well as the number of minority students entering Ph.D. or MD/Ph.D. programs, especially in the biomedical sciences.

The lessons learned from these and other programs are numerous and significant [12–14]. Although there is strong evidence that specialized support programs synergistically increase the number of graduating STEM students not affiliated with scholars programs, both in STEM fields and across all disciplines [15], data show that many promising students either fail to be retained in STEM majors or complete STEM degrees but are not competitive for top jobs or admission into graduate programs [10, 16]. STEM BUILD at UMBC will be drawing from those students who were not qualified to participate in the Meyerhoff, MARC and/or LSAMP scholars programs. This pool of students (i.e., Tier 2) are less likely to graduate or be retained in the STEM discipline of their choice. These data provide the context and motivation for the STEM BUILD at UMBC study.

## STEM BUILD at UMBC

### Program aims and evidence base for initiatives

STEM BUILD at UMBC targets promising Tier 2 students who are likely to benefit from additional mentoring, training, and assistance to overcome the hurdles that are often a result of unfamiliarity with best practices (first generation college), poor advising, financial need (socio-economic factors), or a deficit of preparation/inspiration (traditional classroom lecturing). Most importantly, applied learning experiences are well-known to be transformational to a student’s success (in STEM and non-STEM areas), but access to them is limited by the number of positions available to non-scholars for internships, mentorships, and undergraduate research positions. The practices of STEM BUILD at UMBC are being vetted to increase the number of students from all backgrounds who succeed in STEM (Fig. 1).

UMBC’s six-year overall graduation rate averaged 59.4% for first time, full-time, degree seeking freshmen who entered UMBC from 1999 to 2010 and was 63.3% for the cohort entering in Fall 2010 [17]. Generally, only half of all students enrolled in postsecondary education graduate [16, 18]. Given the nation’s troubling low graduation rates, policymakers and researchers are interested in identifying factors that predict college graduation. Theoretical and empirical approaches for increasing the number of STEM graduates come from four interrelated traditions: (i) general approaches to increasing retention and graduation rates; (ii) general approaches to increasing retention and graduation rates for underrepresented groups; (iii) approaches to increasing STEM retention and graduation rates; and (iv) approaches to increasing retention and graduation rates for underrepresented groups in STEM. The cost of financial aid to increase retention rates for large numbers of additional students is cost prohibitive.

With the goal of serving as a national model in higher education, the STEM BUILD at UMBC is *using research and understanding of best practices* to increase diversity participation and STEM success. Research literature [19] suggests that the critical factors for minority student success, performance, and persistence in STEM fields include: (i) knowledge and skills, (ii) motivation and support, (iii) monitoring and advising, and (iv) academic and social integration. The unique approach of STEM BUILD at UMBC (in contrast to individual scholars programs) is to infuse methods addressing these critical factors, especially the mentoring and training process, into the fabric of undergraduate degrees and experience. The goal of this approach is provide all matriculating STEM students across the academic performance spectrum with the opportunities and advantages of a scholars program (e.g., a simulated one-on-one faculty applied learning experience). Of primary importance are *capacity, scalability, and institutional sustainability* as we envision the advantages and opportunities of individual scholars programs applied to *all* students. Therefore, the STEM BUILD at UMBC motto of “Think 500, not 50!” is a major factor in all program decisions.

While many successful programs are replicated, few use methodology to study program factors that contribute to student success and which can be effectively scaled up for broader impact. Meyers and Dynarski argue that the best method to distinguish between the factors is to randomly assign students to treatment groups [20]. The desire for a study complemented the NIH requirement to include an undergraduate training program with prescribed (i.e., NIH Kirschstein) support. In order to critically assess outcomes, evaluate the success of initiatives, and enhance the potential adoption of components by other institutions, the STEM BUILD training core is being conducted using Randomized Control Trial (RCT) methodology, vide infra. An extensive effort by an experienced assessment team is an essential component of the initiative’s evaluation plan.

### BTP trainees and BUILD affiliates - recruitment

BTP Trainees are the pioneers of this potential national model that answers the call to retain and improve the success of STEM majors, especially those from underrepresented groups. The BTP is being designed for its scalability with the overarching goal of ensuring that the BTP Trainees (as exemplars) are retained in STEM majors and better prepared for future opportunities. Staff work with the lead evaluator and the Office of Financial Aid and Scholarships (OFAS) to identify a pool of first year students (freshmen and transfers) using a Tier 2 recruitment protocol that aligns with NIH expectations for race neutrality and other eligibility requirements for freshmen (i.e., citizenship status, full-time, targeted STEM major, MSAT ≥550 and a minimum HSGPA of 3.0 and typically less then 3.4) and transfer (i.e., earned an associate’s degree or 56 college academic credits with a minimum GPA of 3.0). Identified students are formally invited to apply for the STEM BUILD research study using an online application and understand that, if selected, they may have the opportunity to be placed in one of three groups – Group 1: BTP with significant scholarship and programmatic support and STEM LLC housing; Group 2: Guaranteed campus housing in the popular STEM Living and Learning Community with programmatic but no monetary support; and Group 3: Control/Comparison group with monetary incentives for survey completion. To allow for scalability and ensure that applicants identify with the mission of STEM BUILD at UMBC, the online application includes four essay questions adapted from interview questions used by other UMBC scholarship programs to gauge the mindset of applicants. Selections must be finalized to meet the annual UMBC housing deadline of May 1st.

The Affiliate BUILD Program, *the BUILD a Bridge to STEM Summer Internship*, is the focus of the Research Enrichment core of the STEM BUILD at UMBC Initiative. Students, nominated by their collaborating four-year institution or community college, spend 6 weeks working in BUILD Group Research (BGR) teams to explore real problems with faculty or industry researchers at UMBC. The summer internship, which extends the inclusive alliance of the Gates-funded t-STEM Transfer Student Success Initiative, is intended to be a potential BTP recruitment tool for eligible transfer students.

### BUILD programs description

#### BUILD Training Program (BTP)

The STEM BUILD at UMBC program weaves the many aspects of research into a series of training and mentoring experiences targeting Tier 2 students that build and escalate over a four-year cycle. The program is intended to fit into the standard academic educational pathway of multiple STEM majors. BTP Trainees will need time and additional effort to build their skills, competencies, and confidence, which is what STEM BUILD at UMBC is designed to accomplish. Table 1 outlines the mentoring and training program for the BTP Trainees. The program is divided into four periods that reflect the standard BTP pathway and are described as follows.

**Table 1:** Outline of the mentoring and training program for BTP Trainees

BUILD Period	SEM/YR	BTP Trainees	Comment
1	SR 15	**Build a Bridge to Success (BBS)** Math Preparation SURF – Attendance	*Establishing a strong foundation* Math 106 – Algebra (if needed) **BBS** – Career engagement and exploration, the Scientific Method, Group Research Experience
	FA 15	**Quantitative Reasoning Laboratory** **Intro to a Research University (IRU)** STEM LLC, BUILD UP, Leadership Series UGRS/posters (Microbiome Project)	**IRU** – study skills, time management, academic integrity, communication skills, diversity Pre-Calculus and English 100/110 Standard Classes^a^
	SP 16	STEM LLC, BUILD UP, Leadership Series	Standard Classes^a^
2	SR 16	**Bioanalytical Research Training (BRT)** EHS 115 – Medical Terminology^b^ SURF, (Bridge Repeat)^d^	* Developing technical laboratory skills in context of authentic question*
	FA 16	**Intro to Discovery (ID)** UGRS/Posters (BRT) STEM LLC^c^, BUILD UP, Leadership Series	**ID** – Finding research opportunities, Applications/Resumes/Elevator Pitches Standard Classes^a^
	SP 17	STEM LLC^c^, BUILD UP, Leadership Series	Standard Classes^a^
3	SR 17	**Phage Hunters, REU, Internships, BGR** GRE Preparation, SURF, (Bridge Repeat)^d^	*Engaging in authentic research and developing laboratory, scientific communication, and research skills*
	FA 17	**Intro to Post-Bac (IPB)** UGRS/Posters (Phage Hunters) STEM LLC^c^, BUILD UP, Leadership Series	**IPB** – Careers/Proposal Writing Standard Classes^a^
	SP 18	**Ethics and Integrity in Scientific Research** URCAD/Oral Presentations STEM LLC^c^, BUILD UP, Leadership Series	Standard Classes^a^
4	SR 18	**REU, Internships, BGR** GRE Preparation Course Biomedical Case Studies Course (Bridge Repeat)^d^	*Implementation of Skills and Competencies*
	FA 18	**UGRS/Posters** STEM LLC^c^, BUILD UP, Leadership Series	Standard Classes^a^
	SP 19	**URCAD/Oral Presentations** STEM LLC^c^, BUILD UP, Leadership Series	Standard Classes^a^

^a^As defined by each students' personal pathway plan

^b^Completed at some point during their undergraduate studies

^c^Voluntary participation in the STEM LLC

^d^Summer BUILD Repeat – Start of a new BTP Trainee cohort for each academic year of the program

BUILD Period 1 is designed as a six-week hybrid program (half online and half on-campus) to *establish a strong foundation of basic skills and academic enrichment*. BTP Trainees participate in the BUILD a Bridge to Success Summer program that focuses on intensive preparation of math skills and academic preparedness, leadership, STEM career exploration and planning, a BGR experience (i.e., Microbiome Project), stress-reduction activities, community-building, and exposure to the UMBC research community through attendance at the University’s annual Summer Undergraduate Research Fest (SURF) each August. The BUILD a Bridge to Success Summer program repeats each summer for new Trainees. In the fall and spring, Trainees enroll in classes according to their personal academic pathways, which have been planned with advisors from the BUILD program and the students’ academic majors. All Trainees enroll in the interdisciplinary Quantitative Reasoning: Measurement and Skills Laboratory (two credits) to further develop their quantitative and scientific reasoning skills. Introduction to a Research University (one-credit) is a seminar-style class that focuses on academic and personal success skills (i.e., time management, study skills), presenting research (i.e., Microbiome Project) at a fall regional undergraduate research symposium, and learning about science and research. Beginning in spring, all Trainees participate in the BUILD UP program, which is then scheduled for every semester of a trainee’s tenure in the program and includes a monthly leadership seminar series.

BUILD Period 2 is designed to train students in the *foundational bioanalytical skills needed for a research career, especially concerning data, its collection, and interpretation*. In the second summer, Trainees explore the myriad of molecular biology (e.g., polymerase chain reaction, Western Blot, etc.) and bioanalytical techniques and instruments (e.g., mass spectrometry, nuclear magnetic resonance spectroscopy, chromatography, etc.) needed to conduct biomedical research by participating in Bioanalytical Research Training (BRT). As part of BRT, students learn to select the proper tools/instruments, design controlled experiments, collect measurements, analyze and interpret experimental results, and advance their public speaking skills. During students’ second year, they must complete the Introduction to Research (Discovery) online (zero-credit) practicum, which is designed to help students find research opportunities, explore mentoring styles and the research culture, and develop professional skills needed to communicate with others in the STEM discipline. Before graduation, all Trainees must enroll in EHS 115: Medical Terminology, which is an online course designed to introduce students to the language of medicine and related research careers. Further, each student must complete the one-credit Ethics and Integrity in Scientific Research (BIOL 397) course, which is typically completed during the spring semester of their second year.

BUILD Period 3 is organized to help students further build *confidence and skills associated with performing independent research* through a group research experience specifically modified for BTP Trainees from the introductory UMBC Phage Hunters experience during their third summer [21]. Trainees complete a non-credit experience that represents a condensed version of the first semester, wet-lab, component of the in the HHMI Science Education Alliance – Phage Hunters Advancing Genomic and Evolutionary Sciences (SEA-PHAGES) program (http://www.hhmi.org/science-education/developing-scientists). In that experience they isolate and characterize bacteriophages from soil samples using microbiological and molecular biology techniques [22]. This abridged version provides students with a brief but intense introduction to microbiological and molecular biology-based research. Some students are inspired to matriculate in the full credit-bearing PHAGE experience during the academic year, while others pursue other forms of applied learning experiences. The third summer program will be supplemented with Graduate Record Exam (GRE) preparation sessions, and followed by BUILD UP programming with a practicum entitled Introduction to Post-Bac, focusing on various STEM career paths and the professional development skills needed for a career in biomedical research and proposal writing. All BUILD students are expected to present or attend at the UMBC Undergraduate Research Symposium in the Chemical and Biological Sciences (URS) in the fall and/or the campus-wide Undergraduate Research and Creative Achievement Day (URCAD) in the spring.

BUILD Period 4 is dedicated to *applying skills and competencies* obtained by Trainees during their tenure in this program. Activities during the fourth training year include an approved research experience (e.g., REU, Internship, or BGR project) over the summer and a Biomedical Case Studies course that requires students to practice reasoning and knowledge skills on real-world cases. Trainees will present the results of their research experience for presentation at SURF (Summer), URS (Fall), URCAD (Spring), and/or an appropriate regional or national conference.

Throughout the cycle of the four STEM BUILD periods, trainees are exposed to and trained through the BUILD *escalating* series of academic enrichment, professional development training, and research mentoring activities. Each year, the STEM BUILD at UMBC Initiative ensures that Trainees attend and/or deliver poster or oral presentations at local, regional, or national research conferences.

### BUILD a bridge to STEM summer internship program

Visiting students from 2 to 4 year schools, or BUILD Affiliates, participate in a six-week summer research internship, where participants work in teams to address real questions in biomedical and behavioral research and are mentored by faculty and professional scientists on or off the UMBC campus. BUILD Affiliates are nominated by our six collaborating institutions (three slots per institution), which include five local community colleges and Gallaudet University, a world leader in the education of deaf and hard of hearing students. Their internships include intensive preparation of laboratory skills, critical evaluation of scientific literature through participation in a journal club, development of technical and scientific writing skills, participation in weekly laboratory meetings, STEM career exploration, and professional development opportunities. University deaf interpreters provide accommodations, as necessary. The BUILD a Bridge to STEM internship culminates in a research symposium, where the members of each team give their research findings as oral presentations. The BUILD Affiliates learn while they earn an hourly wage to explore aspects of their mentor’s research, making the experiences mutually beneficial. Overall, the program supports the STEM BUILD at UMBC Initiative by identifying transfer students for BTP recruitment, vetting the BUILD Group Research pedagogy model, and providing abbreviated group research opportunities.

### Institutionalization and sustainability

The institutional changes described above are focused on the general academic enrichment, socialization, and environment of students. Important, yet more difficult tasks, are the training and mentoring in research aspects of a student’s education. In STEM BUILD at UMBC, we are essentially promoting a “flipped” university, where the students’ training, mentoring, and applied research experiences are infused and incorporated into the classroom as a replacement or supplement for the traditional internship or experience in a research laboratory.

STEM BUILD at UMBC involves the design and implementation of a strategically coordinated group of academic and research enrichment opportunities specifically engineered for Tier 2 students to be successful. At the heart of this proposal is an escalating series of BUILD Group Research (BGR) experiences (Table 2) to acclimate, inspire, and train students, who may never have the opportunity to work one-on-one with faculty mentors. BGR experiences are supplemented with sequential professional development, workshops, and training activities (i.e., *BUILD a Bridge to Success, Introduction to a Research University, Introduction to Research (Discovery), and Introduction to the Post-Baccalaureate*), a *Biomedical Case Studies* course, and a one-credit course in *Ethics and Integrity in Scientific Research*. This program is designed for scalability, in that many components can be incorporated into the traditional academic core.

**Table 2:** Escalating Series of BUILD Group Research Experiences

Item	Description
*Level 1: Establishing a strong foundation of basic skills and academic enrichment*	
**BUILD a Bridge to Success -** *trainees learn from analyzing and interpreting a published large data set.*	A group research experience, where students formulate their own research questions on a large metagenomics data set and analyze and interpret the data (Microbiome Project). Students present findings in an oral presentation presented to all BTP Trainees. Students also engage in a journal club to read, interpret, and understand a journal article.
*Level 2: Foundational skills needed for a research career, especially in regards to data, its collection and interpretation*	
**Bioanalytical Instrument Training -** *trainees are trained in instrumental methods of analysis, interact with research mentors, and develop projects that supplement numerous funded research projects*	Through affinity research group-like projects, students will learn to select the proper tools, collect measurements, and reduce them to statistically meaningful results. This experience is an extension of the Quantitative Reasoning Laboratory; in contrast, it uses projects garnered from faculty laboratories that require the development and/or application of bioanalytical methods and instrumentation.
*Level 3: Confidence in performing independent research*	
**PHAGES -** trainees experience authentic discovery – a natural driver of learning	The well-documented HHMI-Science Education Alliance (SEA) Phage Hunters Advancing Genomics and Evolutionary Science (PHAGES) is used as an applied research experience. According to the HHMI-SEA literature, PHAGES offers students: • ownership of a project • an opportunity to present, publish, and contribute to the scientific community • regular milestones to measure progress • authentic scientific discovery
*Level 4: Capstone implementation of skills and competencies*	
**BUILD Group Research (BGR)** - trainees, in groups, will work with assigned faculty mentors on funded research projects	Student groups with similar interests work with an approved mentor on a project associated with the research of that faculty mentor. The work is intended to be presented at local/national meetings and published. In lieu of the BGR, students may substitute an extended independent research project in an approved faculty mentor laboratory.

### STEM Living and Learning Community (LLC)

A significant attribute of scholars programs is that they inherently provide the student with an identity or affiliation with a group (e.g., Meyerhoff Scholar). A similar effect in a scalable fashion is being obtained through the establishment of a STEM Living and Learning Community (LLC) at UMBC. The STEM LLC is a critical component of the BTP, as it is required for two of the three groups in the study. The STEM LLC encourages camaraderie among its residents, exposes them to students pursuing other STEM majors, expands perspectives, offers peer mentoring, encourages participation in applied learning experiences, develops leadership skills, fosters study groups participation, and fosters cultural/artistic appreciation. The 2007 National Study of Living-Learning Programs (NSLLP) found that participants in LLCs had a higher mean score in regard to feeling that they had a smoother academic and social transition to college, and felt a stronger sense of belonging [23]. These results confirm findings from many studies related to LLCs and their enhancement of transition and retention. Specifically, LLC residents experience positive outcomes from faculty and peer interactions, support services, and community programming [24, 25]. Further, students living in LLCs have an easier college transition, higher academic performance and retention rates, increased academic self-confidence, and lower withdrawal and dismissal rates [23–26]. Most relevant are studies revealing that STEM LLCs promise to deepen students’ commitment to a bachelor’s degree in the STEM disciplines [23, 27].

The STEM LLC opened in Fall 2015 as the tenth of a group of residential communities established and supported as collaborative projects of the Office of Student Affairs (Residential Life), the Provost’s Office, and academic partners. While each community is unique, all have the same goal of fostering successful and engaged UMBC students. LLC programs provide experiences that extend from the classroom to the surrounding residential community.

As one of the largest LLC communities at UMBC, the STEM LLC structured activities are enhanced by the involvement of the BTP Peer Mentors, a group of high achieving undergraduates hired and trained to provide academic program support in the STEM LLC and other areas. The BTP Peer Mentors serve as residence-hall based tutors and facilitate study groups, which help STEM LLC residents (to include BTP Trainees) better master course materials by regularly sharing information, knowledge, and expertise about a course in which they are all enrolled. This environment assists students in learning the material in a deeper, more meaningful way. Peer Mentors maintain regular evening hours in the STEM LLC to support student learning by generating good questions, preparing for exams, and clarifying common misunderstandings with course material. Also, the BTP Trainees and other STEM LLC residents form a student advisory board to plan activities focused on STEM. This student involvement develops trainee leadership skills and ensures that students are excited about the opportunities for fellowship and learning that they plan. BTP Trainees and STEM LLC Residents also learn to use available resources at UMBC to monitor their own progress toward STEM degrees, graduate school acceptance, and preparedness for careers in biomedical and behavioral research.

### Developing faculty

*Faculty Needs Assessment* – The Faculty Development Center (FDC), which is supported by the Provost to provide numerous services and resources to support teaching roles at UMBC, conducted a needs assessment among UMBC faculty involved in the STEM foundation courses and research courses for BTP Trainees. After the FDC Staff first facilitated focus groups of selected faculty asking them about specific needs, the collected data were used to create a template to gather data from all faculty teaching foundational STEM courses or proposed research courses at UMBC (e.g., available and needed resources, program, and training). The template documented current practices, areas of programmatic need and targeted improvement (with data-driven rationale), and available and needed resources. The template was analyzed for areas of convergence/divergence. The results are summarized as follows:

- A significant majority of faculty used active learning techniques (e.g., class discussion, real-life problems, and small group work) in all or most of their classes and less likely to use other active learning techniques (e.g., laboratory learning/field learning, reflective writing, and clickers/electronic feedback in class) in all or most of their classes.
- Faculty reported feeling extremely or very confident at academic-oriented tasks, such as setting learning goals, selecting reading materials, designing assignments, planning class activities, and engaging students in learning, but fewer faculty reported feeling extremely or very confident in their abilities to promote student collaboration and address sensitive issues in ways that help students to deal with them maturely.
- In terms of mentorship, faculty reported feeling extremely or very skilled at helping mentees develop strategies to meet goals, building mentees’ confidence, and establishing a relationship based on trust. Fewer faculty reported feeling extremely or very skilled at taking into account the biases and prejudices the mentor may bring to the mentor/mentee relationship and/or discussing diversity issues.
- Faculty strongly agreed or agreed that students can substantially change how much science ability they have and substantially change how much math ability they have. Fewer faculty strongly agreed or agreed that instructors can substantially change how much science ability students have and/or substantially change how much math ability students have.

Based on results from the Faculty Needs Assessment for UMBC faculty, FDC services were integrated into the support of the BUILD project by providing a specialized training program.

*Faculty Training Program for Teaching BTP Trainees* – A specialized Training Program consists of orientation activities, ongoing workshops and one-on-one support to individual faculty. The training program, beginning with the orientation, provides a mechanism for changing departmental cultures and practices. This mechanism not only impacts the students under the BUILD initiative, but promises sustained institutional change going forward. The FDC collaborated with departments offering STEM foundation courses and research courses to design an ongoing series of workshops. The FDC engaged department chairs in crafting aspects of the workshops to maximize their impact. The workshops engage faculty in ideas and activities designed to create classroom and departmental environments most conducive to fostering student feelings of inclusion and self-efficacy, thus increasing their chances for success. Specific topics include training related to implicit bias, cultural competency, stereotype threat, as well as the latest work on the most effective pedagogical practices to promote student learning [28–30]. For example, pedagogical practices that are especially relevant for teaching at-risk students include cooperative learning [31, 32] and team-based learning strategies [33]. These approaches build in clear expectations for student work, foster the development of students’ individual responsibility for learning, provide group support and require group accountability, and build students’ self-efficacy. To provide sustained one-on-one support, faculty who teach in the foundational and research courses that include BTP Trainees were encouraged to invite FDC staff or faculty colleagues trained to provide constructive feedback by observing several class sessions. In addition, faculty had the option to request that the FDC collect midterm feedback to determine how students are experiencing the class in time to make needed corrections before the end of semester. The Training Program is being sustained by an ongoing new certificate program, named Active Learning, Inquiry Teaching (ALIT), designed to support faculty in adopting active learning pedagogical approaches that foster the development of their students as scientists and lead to their academic success [28, 34]. These approaches, informed by the research on learning, help faculty provide all students with deliberate practice in the skills and habits of mind necessary for learning, inquiry, and research. This program is open to all faculty who teach courses in the College of Natural and Mathematical Sciences and the College of Engineering and Informational Technology and is supported by the Colleges, the FDC, and the NIH-funded STEM BUILD at UMBC Initiative. ALIT Program completers receive certificates, letters sent to their chairs and Deans for purposes of promotion or tenure, and professional development stipends of $500 each. The requirements for the two-year certificate program, which are available in detail at https://fdc.umbc.edu/active-learning-inquiry-teaching-certificate-program/, include:

- Attendance at a minimum of eight programs during the first year and four programs during the second year, including a kickoff mini-retreat,
- Participation in the first or second year in a class observation cycle with the FDC,
- Participation in the first year in the FDC’s Classroom Assessment for Teaching And Learning, or CATALyst Process for gathering course midterm feedback,
- Completion of reflective surveys on teaching practices provided by the FDC at the beginning and end of each program year, and
- Help with facilitation of sessions at the retreat or other FDC second-year programming.

From the onset, the goal of STEM BUILD at UMBC has been to elucidate effective practices that promote STEM student success that can be scaled to many students. As noted above, UMBC through the STEM BUILD at UMBC initiative is in the process of institutionalizing many of its central components. The overall impact of faculty professional development on departmental practice and culture is being monitored through faculty surveys and the Coordination & Evaluation Center (CEC) of the NIH BUILD partnership.

### Collaborations and pipeline partnerships

STEM BUILD at UMBC benefits from UMBC’s leverage of existing relationships with its five top feeder community colleges (Anne Arundel Community College, Community College of Baltimore County, Howard Community College, Montgomery College, and Prince George’s Community College), the University of Maryland, School of Medicine (UMSOM), and Gallaudet University. Using an approach termed *Collateral Synergies* to improve student success for a broad range of students, these seven institutions agreed to participate in STEM BUILD as collaborators, not paid partner sub-awardees, as part of an ongoing “inclusive alliance” with UMBC to support student success. As previously mentioned, our community college collaborators along with Gallaudet University support the BUILD a Bridge to STEM Summer Internship and are encouraged to promote the BTP to Associate’s Degree awardees who plan to transfer to UMBC. The UMSOM collaboration, which is evolving, epitomizes the NIH BUILD program goals to inspire students to pursue post-baccalaureate careers in biomedical and behavioral research. In addition to helping with career exploration, planning, and advisement for those interested in medical careers, UMSOM will assist in course development and provide applied research opportunities for select BTP Trainees.

## Site level evaluation design

### Evaluation aims

The STEM BUILD program evaluation is based upon the comprehensive approach used with UMBC’s Meyerhoff Program [6, 10]. The key aims are to: 1) conduct rigorous analyses to examine the *added value* of the BUILD program, by comparing primary academic outcomes of BUILD students to comparison sample students; 2) examine the extent to which theory-based STEM success predictor variables are related to BUILD student academic outcomes, and also compare BUILD students with comparison samples on these measures; and 3) provide formative assessment information to the BUILD Program leadership related to aspects of the BUILD intervention components.

### Key features of the evaluation

The primary study design is a controlled trial, with students being randomly assigned to BTP, LLC and control conditions. Additional comparison samples include an historical comparison sample of comparable students based on propensity score matching and students at peer institutions. Primary academic outcome measures include retention in a STEM discipline and overall and STEM-specific GPA. Key theory-based psychosocial variables assessed (Hallmarks of Success) include research participation, research self-efficacy, and identity as a scientist. Baseline and yearly spring semester surveys are being used to obtain the psychosocial measures. Demographic and pre-college academic variables will be included in statistical analyses. The design also includes a focus on faculty and institutional level outcomes and use of focus groups, interviews and surveys as formative evaluation tools to enhance program development and effectiveness.

## Conclusion: contributions and challenges

STEM BUILD at UMBC brings together years of experience with preparing underrepresented groups for STEM careers, the lessons learned from numerous studies and programs, a reputation for on campus culture change, and evolving new partnerships with regional colleges and feeder schools. We are “building” a scholarly community with the help of the NIH-funded BUILD program. We represent a bold approach, in that a change in faculty mindset and institutional culture will need to occur to achieve the desired goal of providing the opportunity for all STEM students to experience the opportunities and advantages of a scholar program. The grand vision is to eventually incorporate as much of this program activity into the academic core of standard pathways to a STEM degree. That is why we have tailored this program to:

- Exploit the existing and strategic infrastructure of the university, whenever possible,
- Develop/redesign courses that complement and can be incorporated into the standard academic core that have the attribute of scalability,
- Add supplemental instruction as add-ons to existing courses to decrease course burdens on students,
- Establish a STEM Living Learning Community that is sustainable, and
- Explore and test BUILD Group Research as a means to reach the many students who are shut out of the traditional one-on-one applied learning experiences.

The purpose of this document is to provide a vision/scaffold on which to build a foundation with the potential to provide a significant number of students with a scholar-like experience as part of their academic program. New courses, supplemental instruction, a STEM Living Learning Community, and BUILD Group Research experiences are all scalable and sustainable (through resource allocations) if these interventions are confirmed to have a significant impact on student success by the Randomized Control Trial (RCT). Faculty buy-in is dependent on this same data and the will of the institution to support change.

The STEM BUILD at UMBC initiative is beginning to make a significant impact to the learning infrastructure for STEM students. Institutionally, the depth of experience and expertise in regard to traditional “high touch and high expense” scholars programs is significant. STEM BUILD at UMBC is attempting to improve the performance of students in the central portion of the academic performance curve by working with promising students who lie below the accepted standards for entrance into scholars’ programs. The challenge is that the inherent expertise, pressure from Trainees, and expectations of external purveyors continuously pulls in the direction of transforming the BUILD Training Program into a scholars program. It is imperative that these biases be overcome if we are to live up to our motto of “Think 500, not 50!” to fulfill both the needs of our nation in producing a million more STEM professionals and the dreams of students to become practicing scientists.
